# Can Hot Weather Cause Non-ST Elevation Myocardial Infarction in Young Athletic Male Runners?

**DOI:** 10.7759/cureus.42936

**Published:** 2023-08-04

**Authors:** Islam H Elrobaa, Ahmed Elmaasarawi

**Affiliations:** 1 College of Medicine, Qatar University, Doha, QAT; 2 Emergency Medicine, Hamad Medical Corporation, Doha, QAT

**Keywords:** reassessment, observation, cardiac attack, hot weather, athlete

## Abstract

Athletes are a group of people who have good activity, regular muscular exercise, an almost standard lifestyle, and should be in good condition with low rates of medical, particularly cardiovascular, complications. However, cardiac attacks, sudden cardiac deaths, and fatal arrhythmogenic syndromes have been reported in athletes with low incidences. We can determine external and internal factors that lead to cardiac attacks in athletes. The former include abnormal cardiac structures and genetics, while the latter include environmental conditions like extreme temperatures, smoking, and drug abuse. Here, we report a case of a cardiac attack with non-ST elevation myocardial infarction (NSTEMI) in a young athlete who was a non-smoker, did not have any abnormal heart structures or a history of drug abuse, and did not have a family history of cardiac disease or arrest. High humidity levels and temperatures were the main causes of the cardiac attack, which occurred during a sports exercise at high temperatures with high humidity levels. We hope to prevent the recurrence of such a case. We need to understand when and where sports exercises can be performed without the risk of medical complications.

## Introduction

There is a low incidence of sudden cardiac attacks in athletes [1]; these incidents may elicit a dramatic response in the media. Athletes should be in good health without medical complications; however, some athletes have reported cardiac attacks. Sudden cardiac arrests and fatal arrhythmias have been reported in athletes. This could be a result of genetic causes that lead to abnormal cardiac structure or other external causes, such as drug abuse or bad weather. Severe hot weather may negatively affect the cardiovascular system because it leads to severe dehydration and acid-based disturbances. Here, we report a case of non-ST elevation myocardial infarction (NSTEMI) in a young athlete during extremely hot weather with high humidity. The patient had no abnormal cardiac structure and no family history of heart disease, cardiac arrest, diabetes, smoking, or drug abuse. This was his first time running at a high temperature and high humidity, similar to our area. We suggest that environmental conditions may be the cause, as no other risk factors were detected. Additionally, some studies have suggested that hot weather induces acute coronary syndrome.

## Case presentation

**Figure 1:**
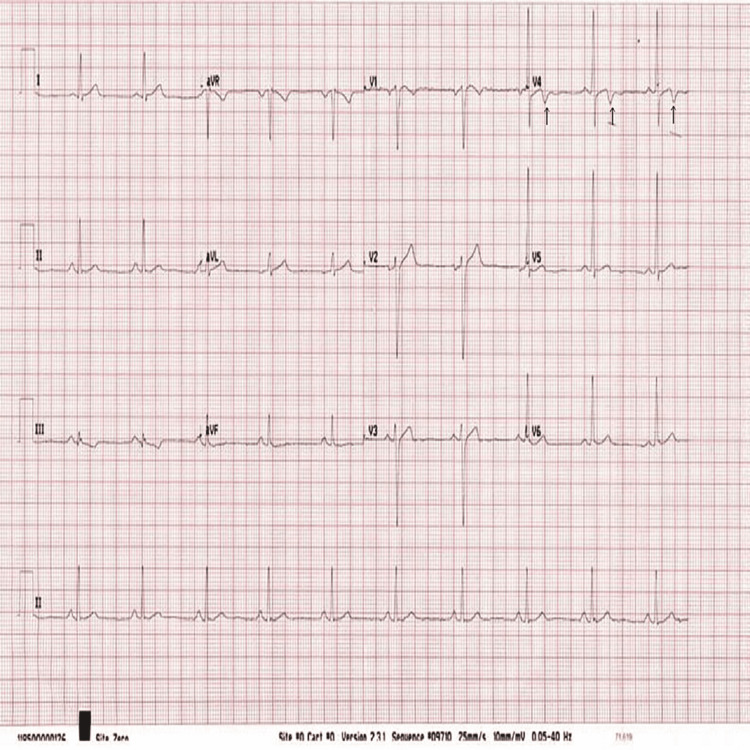
The first ECG showed significant biphasic T wave at V4 and non-significant inverted T in lead III and V1

**Figure 2:**
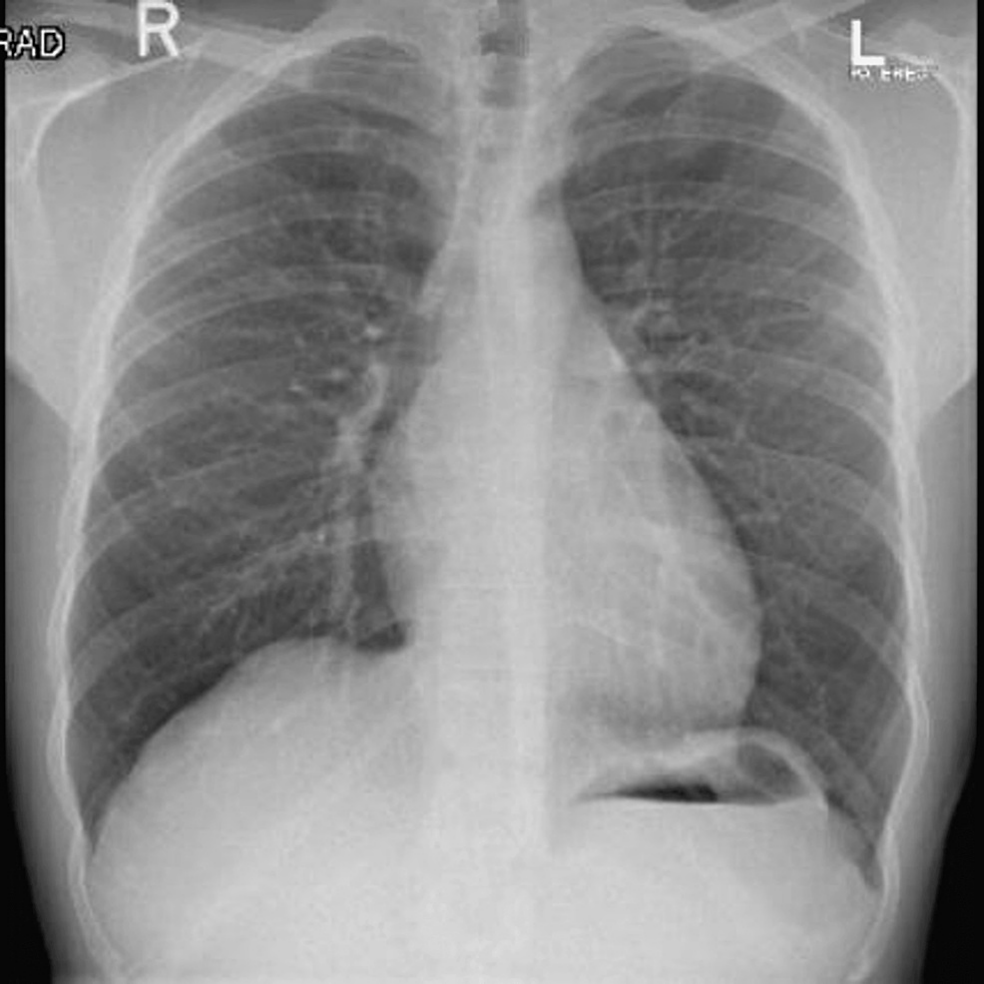
Chest X-ray with normal variation

**Figure 3:**
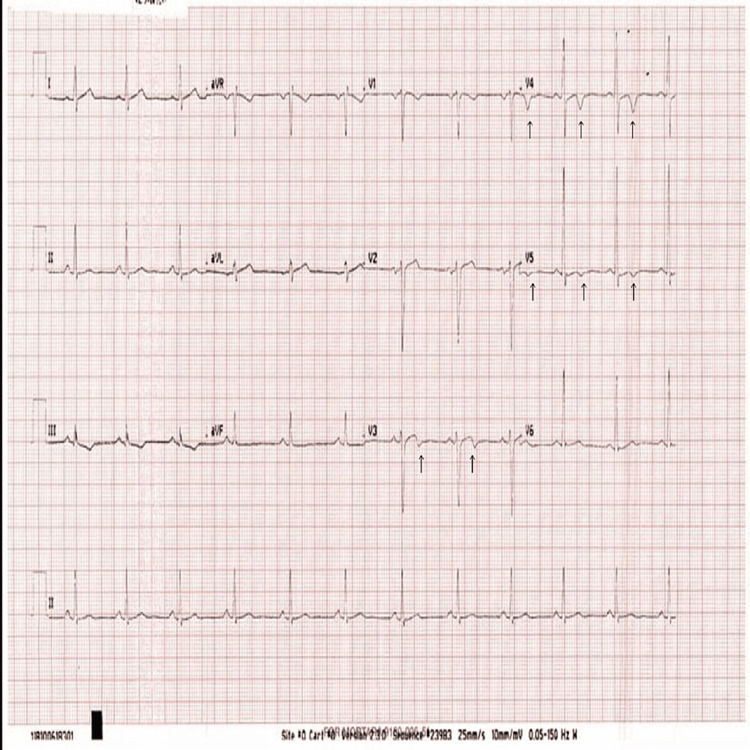
The second ECG showed significant biphasic T wave at V3, 4, and 5

**Figure 4:**
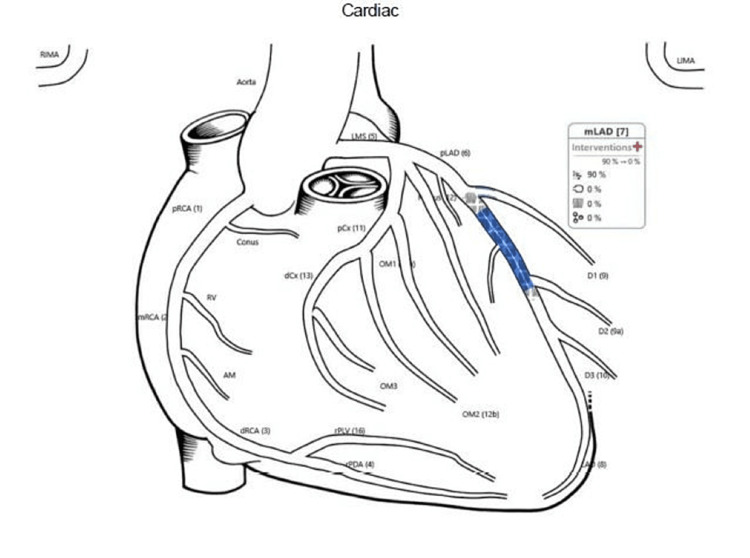
Diagram explained the coronary intervention for our patient

The patient was a 39-year-old male athlete. He denied having any medical complications and had no family history of cardiac disease. He was a runner and usually ran approximately 10 to 15 km per day. He came to Doha, Qatar, in the summer to run in the hot weather. Our patient was from a developed country in the north, and it was his first time running in hot weather with high humidity, as in our region. On the first day of running in Doha, he ran only 2.5 km before experiencing dizziness, severe breathlessness, and palpitations. He presented to our emergency department by ambulance, and his electrocardiogram (ECG) showed a significant biphasic T wave in V4 (Figure 1). Blood tests showed a mild elevation in troponin T to approximately 51 ng/L (normal up to 14 ng/L). His chest X-ray had no abnormalities detected (Figure 2). After four hours, the troponin T level fell to 43 ng/L, and four hours thereafter, the troponin T level fell to 31 ng/L. The patient had a normal level of renal function and creatine kinase (CK). His D-dimer showed negative results. Subsequently, the patient experienced chest discomfort with a breathless sensation again while going to the bathroom. The patient had normal oxygen saturation and heart rate. His ECG showed significant biphasic T waves in V3, 4, and 5 (Figure 3), which were new changes. The patient was admitted to the cardiology department. Echocardiography revealed no regional wall motion abnormalities. Coronary cardiac computed tomography (CT) showed an angiography calcium score of 61.8 (from 11 to 100 means mild risk of coronary artery disease) and mild narrowing of the Proximal RCA. Cardiac angiography revealed single-artery disease in the left anterior descending (LAD) proximal LAD with 90% long stenosis. Therefore, the patient underwent percutaneous coronary intervention in the LAD using a drug-eluting stent (Figure 4). The patient’s condition improved, and he was discharged after a few days.

## Discussion

The acute coronary syndrome is considered an unexpected event in young athletes and is not a common cause of cardiac attacks in athletes [1]. Emergency medicine physicians should be aware of unexpected diagnoses as part of the differential diagnosis. Cardiovascular disease is very rare in athletes who run 10-15 km daily. However, the incidence of cardiovascular attacks has been reported in some athletes [2]. Almost all cardiovascular attacks have been reported in athletes, including fatal arrhythmias, sudden cardiac arrest, and death [3]. This may be a genetic cause resulting in an abnormal heart structure in the coronary artery, left ventricular hypertrophy, hypertrophic cardiomyopathy, or valvular disease [4]. Commotio cordis, an ion channelopathy, is suggested when autopathy remains undiagnosed [5].

The acute coronary syndrome may be rare in athletes but can occur with coronary artery anomalies, atherosclerosis, or drug abuse [6]. Some studies have suggested a relationship between extreme temperatures and coronary artery syndrome, particularly at severely hot temperatures [7]. Some studies have reported an increased rate of acute coronary syndrome admissions in general hospitals during severe hot weather [8]. Other studies have suggested considering extreme temperatures as a risk factor for acute coronary syndrome in order to decrease and prevent it during these weather conditions [9]. Hypercoagulability may increase during hot weather because of the elevated body temperature [10]. In athletes, we can determine the sequence of events that leads to hypercoagulability in hot weather, which induces acute coronary syndrome. Hot weather induces the loss of body fluids and dehydration. Dehydration can cause acid-base disturbance, electrolyte imbalance, and large adrenergic secretion during exercise, all of which lead to increased hypercoagulability and an increased risk of acute coronary syndrome [5,10].

In this case, the patient was an athlete who usually ran 10-15 km daily. Our patient practiced in a developed country in the north; therefore, his exercises were performed under different weather conditions. On the first day in our region, he ran only 2.5 km before experiencing acute coronary syndrome symptoms. High temperatures and humidity play significant roles in these events. Severe dehydration, acid-base disturbance, and electrolyte imbalance with adrenergic secretion during exercise can cause hypercoagulability and coronary block. For the FIFA World Cup in 2022, in Qatar during November and December 2022, the weather was significantly different from that during the summer period from June to September. Therefore, it was appropriate weather for big sporting events.

At our hospital, we considered the possibility of acute coronary syndrome as part of the differential diagnosis in this case. We performed serial troponin T and ECGs. The decrease in troponin T levels may reflect a low possibility of acute coronary syndrome; however, during the observations, the patient had symptoms of acute coronary syndrome with ECG changes; thus, the patient was admitted to the cardiology department. Angioplasty led to the definitive diagnosis of his coronary problems. Clinicians should consider observation, reinvestigation, and reassessment to reach a proper diagnosis; they should not depend only on the primary result.

## Conclusions

We report NSTMI in an athletic patient without a medical or cardiac family history. The patient was not a smoker or alcoholic and had no risk factors for cardiac disease. Hot weather and high humidity may induce heart attacks in athletes. Reassessment with repeated ECGs was the key to diagnosing the case when we found ECG changes in the chest leading to recurrent symptoms of chest discomfort and shortness of breath. Therefore, reassessment, observation, and repeated ECGs are important for diagnosing acute coronary syndrome, even if the patient has decreased Trop T levels.
